# Jagged1 intracellular domain modulates steroidogenesis in testicular Leydig cells

**DOI:** 10.1371/journal.pone.0244553

**Published:** 2020-12-30

**Authors:** Sudeep Kumar, Hee-Sae Park, Keesook Lee

**Affiliations:** School of Biological Sciences and Technology, Chonnam National University, Gwangju, Republic of Korea; National Institute of Child Health and Human Development (NICHD), NIH, UNITED STATES

## Abstract

Leydig cells represent the steroidogenic lineage of mammalian testis, which produces testosterone. Genetic evidence indicates the requirement of Notch signaling in maintaining a balance between differentiated Leydig cells and their progenitors during fetal development. In primary Leydig cells, Notch1 expression decreases with testicular development, while the expression of its ligand, Jagged1, remains relatively unchanged, suggesting that the roles of Jagged1 extend beyond Notch signaling. In addition, Jagged1 is known to be processed into its intracellular domain, which then translocate to the nucleus. In this study, we investigated the effect of Jagged1 intracellular domain (JICD) on steroidogenesis in Leydig cells. The independent overexpression of JICD in MA-10 Leydig cells was found to inhibit the activity of cAMP-induced Nur77 promoter. In addition, JICD suppressed Nur77 transactivation of the promoter of steroidogenic genes such as *P450scc*, *P450c17*, *StAR*, and *3β-HSD*. Further, adenovirus-mediated overexpression of JICD in primary Leydig cells repressed the expression of steroidogenic genes, consequently lowering testosterone production. These results collectively suggest that steroidogenesis in testicular Leydig cells, which is regulated by LH/cAMP signaling, is fine-tuned by Jagged1 during testis development.

## Introduction

Leydig cells represent the steroidogenic lineage in the testis and drive male-specific development of germ cells and secondary sex differentiation by producing the male sex hormone testosterone. Progenitor cells in the developing testis, whose population is maintained by active Notch signaling, give rise to fetal Leydig cells. Notch signaling restricts fetal Leydig cell differentiation by promoting progenitor cell fate. The inhibition of Notch signaling or deletion of its downstream target, Hes1, results in an increase in the number of mature Leydig cells [1]. The Notch1 ligand, Jagged1, is also an important regulator that maintains fetal Leydig progenitors in the undifferentiated state, which is guided by the level of interstitial testosterone [2].

Notch is a transmembrane receptor known to mediate intercellular communication required for cell fate determination, stem cell maintenance, and differentiation [3]. The mammalian Notch family includes four Notch receptors (Notch1-Notch4), which interact with several ligands such as delta-like 1 (Dll1), Dll3, Dll4, Jagged1, and Jagged2. Subsequent to ligand binding, Notch is activated by γ-secretase-dependent proteolysis to release the Notch intracellular domain (NICD) [4–6]. The translocation of NICD into the nucleus and association with the constitutive DNA-binding protein, CSL, activates the transcription of downstream targets Hes1, Hes5, and Hes7 as well as Hey1, Hey2, and HeyL [7–10]. Jagged1 is a well-known ligand of Notch receptors. In addition, Jagged1 is processed into the Jagged1 intracellular domain (JICD) sequentially by α-secretase and presenilin/γ-secretase action. This leads to the nuclear translocation of JICD as a signaling fragment, thereby triggering signal responses for Notch inhibition [11]. Jagged1 is also a transcriptional target of Notch3 and contributes to its transcriptional autoactivation in lymphoma cells [12]. Several recent studies have reported the Notch1-independent activities of Jagged1 [13–16].

Various transcription factors regulate the expression of steroidogenic genes [17]. Nur77, an orphan nuclear receptor, which is also known as NR4A1, NGFIB, TR3, and NAK1, is a major transcription factor that controls steroidogenic gene expression in Leydig cells [17, 18]. Nur77, along with Nurr1 and Nor1, are members of the NR4A family of nuclear receptors [19]. There are three functional domains in Nur77, namely, the N-terminal transactivation (activation fragment-1, AF-1), DNA binding, and C-terminal ligand-binding domains. The ligand-binding domain contains another transactivation domain (activation fragment-2, AF-2) [20, 21]. Nur77 binds as a homo- or hetero-dimer to the Nur77 response element (NurRE) and as a monomer to the NGF1-B response element (NBRE) [22, 23]. LH regulates steroidogenesis in Leydig cells and induces the expression of Nur77 [24], which, in turn, regulates the expression of multiple steroidogenic genes including steroid 21-hydroxylase, 20α-hydroxysteroid dehydrogenase, and 17α-hydroxylase (*P450c17*) [17, 25, 26]. Previous studies have reported Nur77 binding at a defined region within the promoters of rat P450c17 [17], mouse steroidogenic acute regulatory (StAR) [27], and human 3β-hydroxysteroid dehydrogenase (3β-HSD) type 2 [28]. Recent studies suggest Jagged1 to be an angiogenic target of Nur77 [14].

In the present study, we investigated the role of Jagged1 in testicular Leydig cells. The expression of Notch1 in Leydig cells decreased substantially with testis development, while that of Jagged1 remained partly unchanged. cAMP-mediated stimulation of mouse primary Leydig cells showed an inverse relationship between the expression of Nur77 and Jagged1. Furthermore, Jagged1 intracellular domain (JICD) overexpression in primary Leydig cells was found to downregulate the expression of steroidogenic genes and decrease testosterone production. Altogether, these results suggest that Jagged1, beyond its conventional role in Notch signaling, is involved in modulating testicular steroidogenesis.

## Results

### Differential expression of Notch signaling genes during testis development

**Fig 1:**
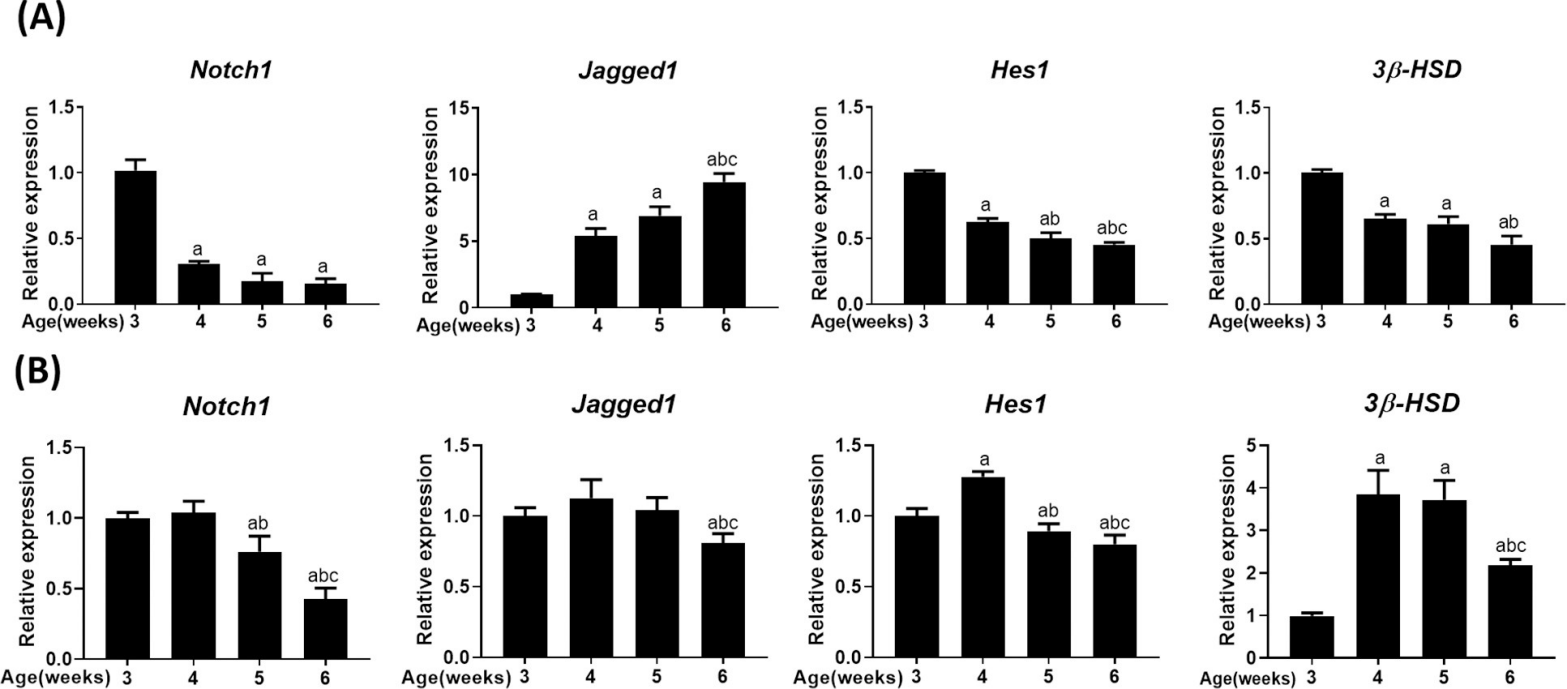
Expression of Notch signaling genes in mouse testis. Expression of *Notch1*, *Jagged1*, *Hes1*, and *3β-HSD* in the whole testis (A) and primary Leydig cells (B) isolated from the testis of mice at the indicated age, as quantified by real-time PCR. The mRNA expressions were normalized to β-actin expression. Statistical significance was confirmed using one-way ANOVA with Tukey’s significant difference (P<0.05) for three independent experiments combined together. The letters a, b, and c denote significant difference relative to 3, 4 and 5 weeks, respectively.

Notch signaling genes, such as *Notch1*, *Hes1*, and *Jagged1*, were expressed in the testis, with their expression being differentially regulated during testis development. The expression of *Notch1* and its target gene *Hes1* was downregulated, while that of its ligand *Jagged1* was upregulated (Fig 1A). However, this expression pattern represents the whole testis, which includes different types of testicular cells. Therefore, we investigated the expression patterns specific to Leydig cells by using primary Leydig cells isolated from the testis at the indicated age (Fig 1B). Although *Notch1* expression pattern showed a slight change, the overall trend of decreasing expression with testis development was maintained. However, the expression patterns of *Jagged1* and *Hes1* were clearly different. *Jagged1* expression appeared almost unchanged, with a partial decrease at 6 weeks. Therefore, the steep rise in *Jagged 1* expression in the whole testis clearly stems from the contribution of germ cells, as previously reported [29]. Similarly, *Hes1* expression remained almost constant along with a subtle reduction with testis development, except the increase observed at 4 weeks. Contrastingly, while *3β-HSD* expression in primary Leydig cells showed a substantial increase during puberty, its expression in the whole testis decreased due to a concomitant decrease in the relative proportion of Leydig cells (compared to germ cells) during testis development.

### Expression of Jagged1 in cAMP-stimulated Leydig cells

**Fig 2:**
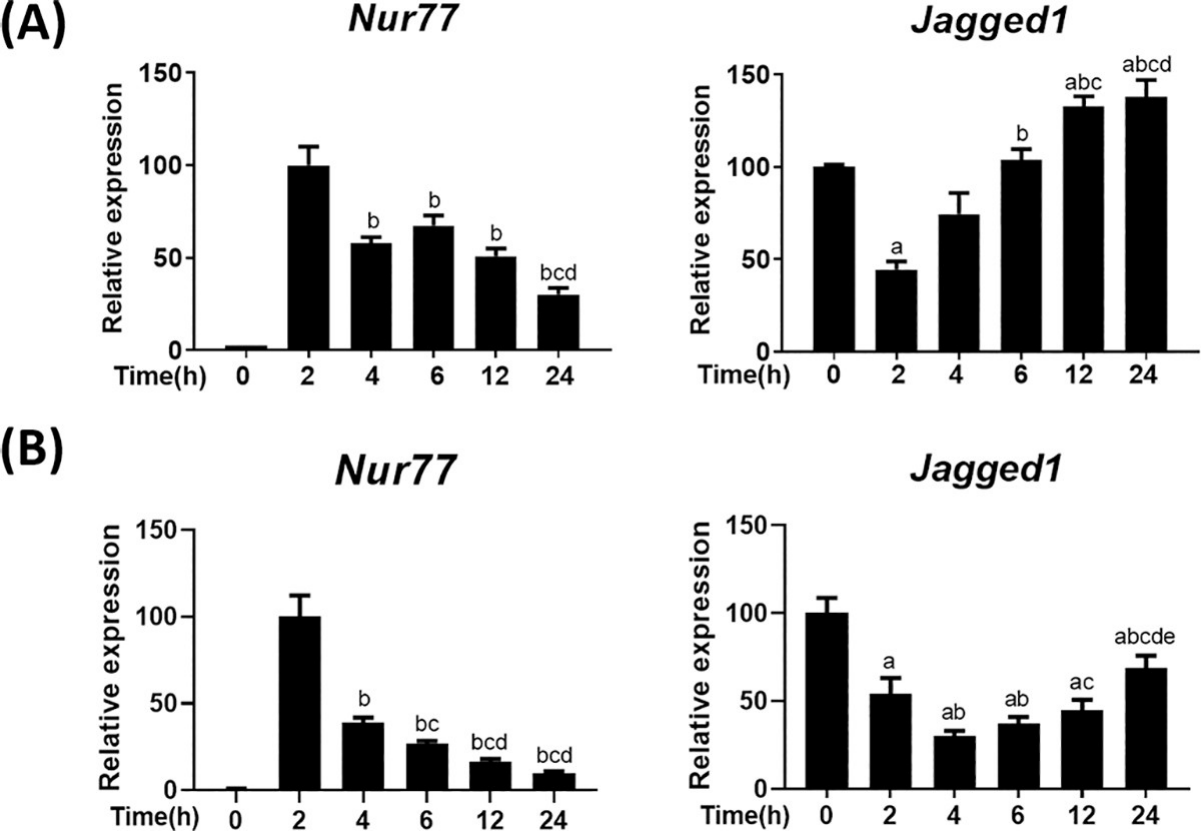
*Jagged 1* and *Nur77* expression shows an inverse relationship. Expression of *Jagged1* and *Nur77* at different time points after cAMP (200 μM) induction in primary Leydig cells isolated from 6-week-old mice (A) and MA-10 cells (B), as quantified by real-time PCR. *Nur77* expression at 2 h and Jagged1 expression at 0 h were considered 100% for relative comparison. Both Nur77 and Jagged1 expression were normalized to β-actin. Statistical significance was confirmed using one-way ANOVA with Tukey’s significant difference (P<0.05). The letter a, b, c, d, and e denote significant difference relative to 0 h, 2 h, 4 h, 6 h, and 12 h, respectively.

Leydig cells initiate steroidogenesis by expressing steroidogenic genes upon induction by LH/cAMP signaling. To investigate the responsiveness of Jagged1 expression to LH/cAMP signaling, we stimulated primary Leydig cells isolated from adult mice with cAMP for different time periods and compared the expression of *Jagged1* with that of *Nur77*, a major transcription factor that regulates the expression of steroidogenic genes, across the experimental conditions. *Jagged1* expression was strongly repressed at 2 h, restored by 6 h, and showed a subtle increase by 12 h (Fig 2A). Meanwhile, *Nur77* expression was strongly induced at 2 h and decreased thereafter. Compared to primary Leydig cells, the time-dependent decrease in cAMP-induced *Nur77* expression was greater in MA-10 cells (Fig 2B). MA-10 cells also showed ~50 and 70% decrease in *Jagged1* expression by 2 and 4 h, respectively. The restoration of *Jagged1* expression in MA-10 cells was delayed and restored to 75% of the original level by 24 h. This repression and induction pattern of *Jagged1* could be inversely related to the marked elevation in *Nur77* expression at 2 h and subsequent reduction till 24 h, which indicates that the regulation of *Jagged1* and *Nur77* is possibly interconnected.

### JICD represses the activity of cAMP-induced Nur77 promoter and its target steroidogenic promoters

**Fig 3:**
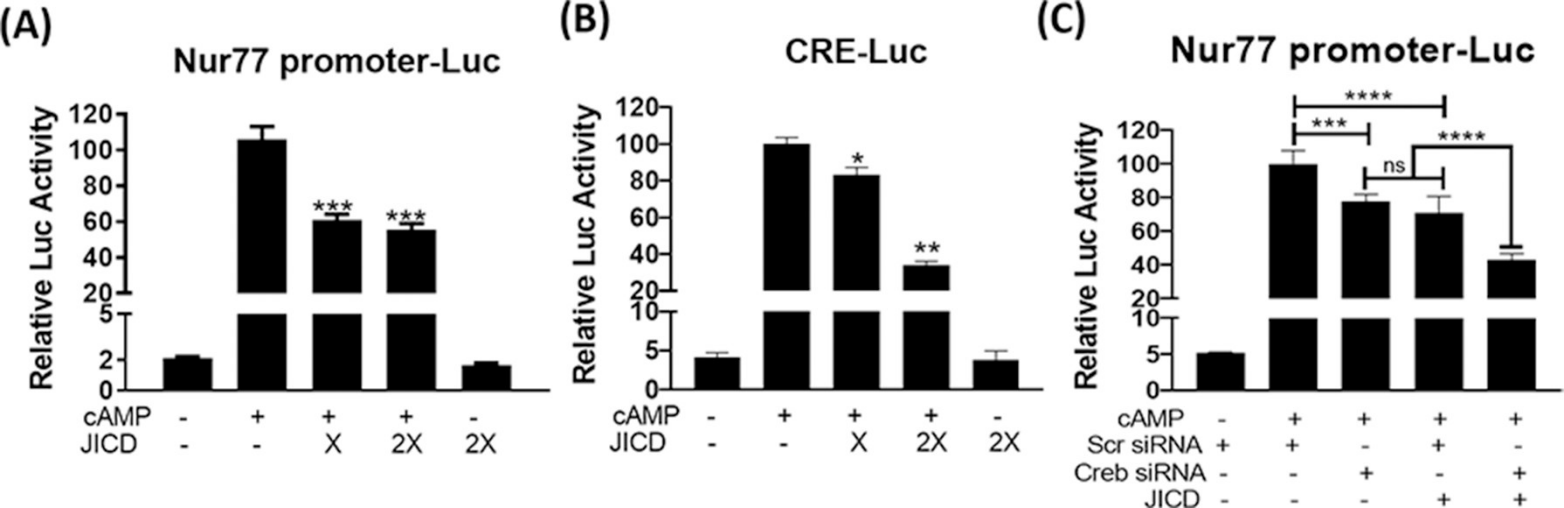
Jagged1 intracellular domain (JICD) represses CREB-responsive Nur77 promoter activity. JICD represses cAMP-induced Nur77 promoter activity (A); CREB-responsive promoter is repressed by JICD in a dose-dependent manner (B); and Nur77 promoter activity is decreased by CREB knockdown using siRNA in the presence and absence of JICD overexpression (C). All experiments were performed using MA-10 cells transfected with the indicated constructs. For determining statistical significance, t-test was performed relative to cAMP-induced controls. *, P<0.05; **, P<0.01; ***, P<0.001; ****, P<0.0001.

Jagged1 is reportedly processed into the membrane-bound and subsequently nucleus-localizing JICD. Nucleus-localizing proteins can regulate the expression of certain genes by interacting with transcription factors. Further, the inversely related expression patterns of *Jagged1* and *Nur77* in cAMP-stimulated Leydig cells allowed the exploration of the effect of JICD on Nur77 promoter activity. Transient transfection analysis revealed the suppression of cAMP-induced Nur77 promoter activity by JICD overexpression in MA-10 cells (Fig 3A). Nur77 expression is regulated by various extracellular stimuli, and certain transcription factors that mediate this regulation have been identified [30]. Among them, cAMP response element-binding protein (CREB) has been reported to regulate Nur77 expression upon cAMP induction in Leydig cells [31–33]. Therefore, we assessed the effect of JICD on CREB transactivation using a CRE-Luc reporter. JICD inhibited cAMP-activated CREB transcriptional activity in a dose-dependent manner (Fig 3B). JICD-mediated CREB inhibition was further confirmed with respect to the Nur77 promoter using CREB siRNA. CREB siRNA repressed the activity of Nur77 promoter similar to the effect exerted by JICD overexpression (~ 25%) (Fig 3C). There was an additive effect when both CREB siRNA and JICD were transfected. These results suggest that JICD represses CRE-dependent activation of Nur77 promoter.

**Fig 4:**
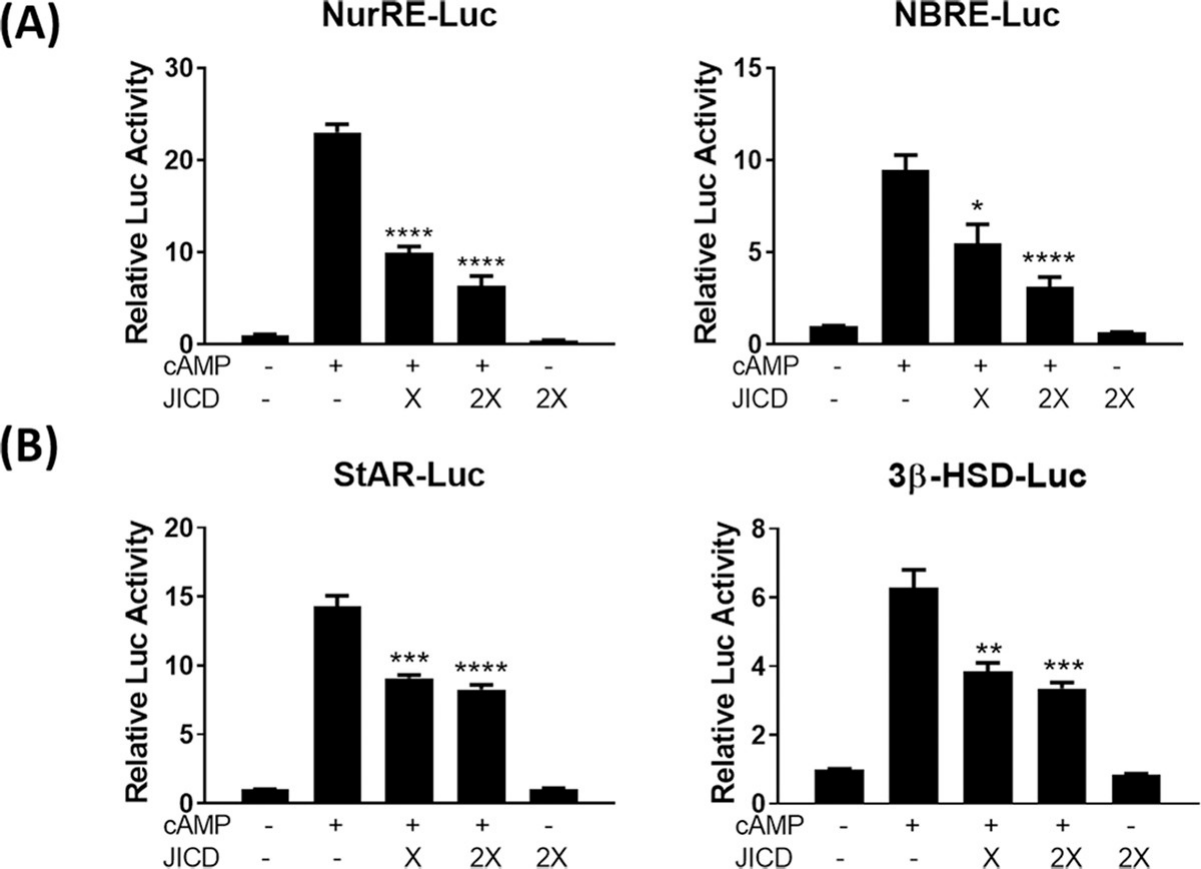
Jagged1 intracellular domain (JICD) regulates the activity of steroidogenic gene promoters. Strong repression of the expression of Nur77-target NBRE and NurRE promoters (A) and downregulation of the expression of Nur77-target steroidogenic gene promoters (B) by JICD overexpression in cAMP-stimulated Leydig cells. All experiments were performed using MA-10 cells transfected with the indicated constructs. Statistical significance was evaluated using t-test performed relative to cAMP-induced controls. *, P<0.05; **, P<0.01; ***, P<0.001; ****, P<0.0001.

The downregulation of Nur77 expression due to JICD-mediated inhibition of Nur77 promoter activity in Leydig cells would decrease the activity of Nur77-target promoters. As expected, JICD overexpression resulted in the repressed activity of Nur77-target promoters, such as NurRE- and NBRE-containing promoters (Fig 4A) and steroidogenic promoters including StAR and 3β-HSD (Fig 4B), in cAMP-stimulated MA-10 cells.

### JICD represses Nur77 transactivation

**Fig 5:**
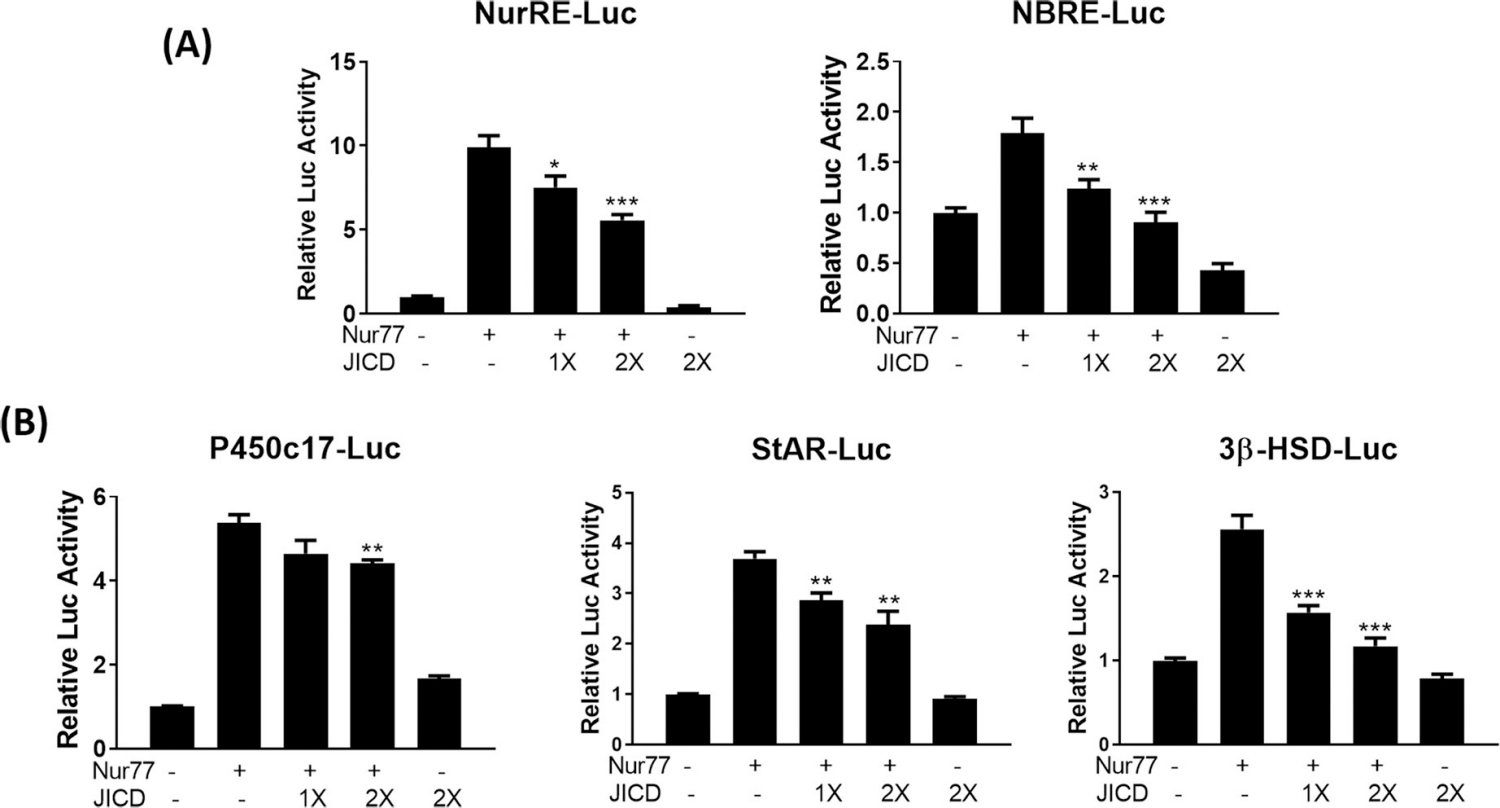
Jagged1 intracellular domain (JICD)-mediated repression of Nur77 transactivation in steroidogenic gene promoters. Repression of Nur77 transactivation in Nur77-target NBRE and NurRE promoters (A) and steroidogenic promoters (B) by JICD overexpression in a dose-dependent manner. All experiments were performed using MA-10 cells transfected with the indicated constructs. For determining statistical significance, t-test was performed relative to cAMP-induced controls. *, P<0.05; **, P<0.01; ***, P<0.001.

Although JICD-mediated repression of Nur77-target promoters might be due to the downregulation of Nur77 expression induced by JICD in cAMP-stimulated Leydig cells, JICD could also directly interfere, in part, in the transactivation of Nur77. To investigate this possibility, we overexpressed JICD along with Nur77 in MA-10 Leydig cells, in lieu of cAMP-mediated stimulation. Interestingly, JICD overexpression inhibited Nur77-induced Nur77-target promoters, such as NurRE, NBRE, P450c17, StAR, and 3β-HSD, in a dose-dependent manner (Fig 5A and 5B). These results suggest that JICD can independently repress Nur77 transactivation, resulting in the downregulation of steroidogenic genes in testicular Leydig cells.

### JICD downregulates the expression of steroidogenic genes in primary Leydig cells

**Fig 6:**
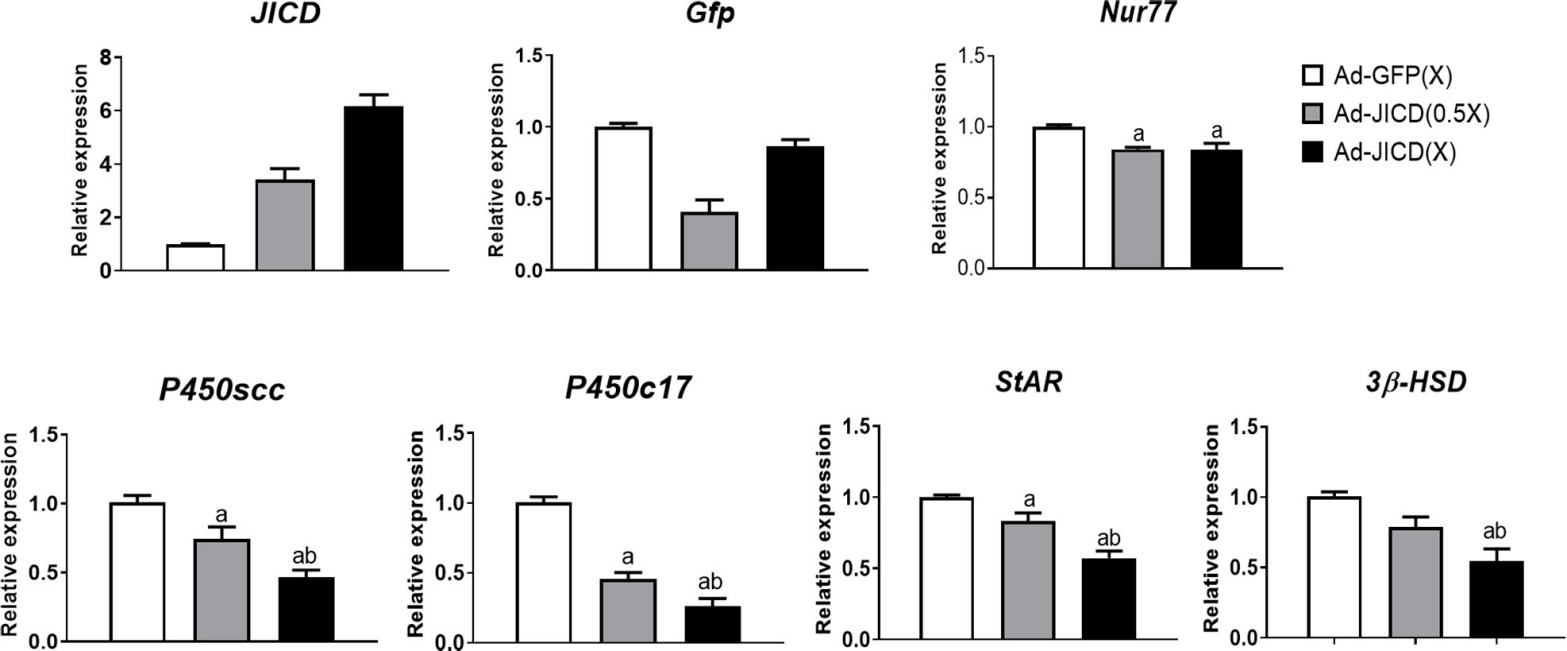
Jagged1 intracellular domain (JICD) represses the expression of steroidogenic genes in mouse primary Leydig cells. Primary Leydig cells isolated from 5–6 weeks old mice were infected with GFP-expressing adenovirus (Ad-GFP) or JICD-expressing adenovirus (Ad-JICD) and stimulated with 200 μM cAMP for 4 h. Adenovirus-mediated overexpression of JICD repressed *Nur77* expression partially, but it strongly inhibited the expression of Nur77-target steroidogenic genes in a dose-dependent manner. The expression of all genes was quantified by real-time PCR and normalized to GAPDH. Ad-GFP-transduced sample was considered the control. Statistical significance was confirmed using one-way ANOVA with Tukey’s significant difference (P<0.05). The letter a and b denote significant difference relative to Ad-GFP(X) and Ad-JICD (0.5X), respectively.

JICD, as we identified, represses the activity of Nur77 promoter, and therefore, it exerts a similar action on its target gene promoters. Furthermore, JICD represses the transactivation of Nur77, resulting in a dual repressive effect on Nur77-target promoters. To confirm the action of JICD in testicular Leydig cells, we infected primary Leydig cells isolated from adult mice with JICD-expressing adenovirus (Ad-JICD), thereby analyzing the effect of JICD on the expression of endogenous steroidogenic genes. Consistent with the results of the promoter-reporter assays in MA-10 cells, the expression of steroidogenic genes, such as *P450scc*, *P450c17*, *StAR*, and *3β-HSD*, were significantly decreased in a dose-dependent manner in Ad-JICD-infected cells compared to cells infected only with the GFP-expressing adenovirus (Ad-GFP) (Fig 6). However, the repression of *Nur77* expression due to JICD overexpression was minimal. These results suggest that JICD represses the expression of steroidogenic genes in Leydig cells predominantly by directly inhibiting Nur77 transactivation.

### JICD modulates steroidogenesis in testicular Leydig cells

**Fig 7:**
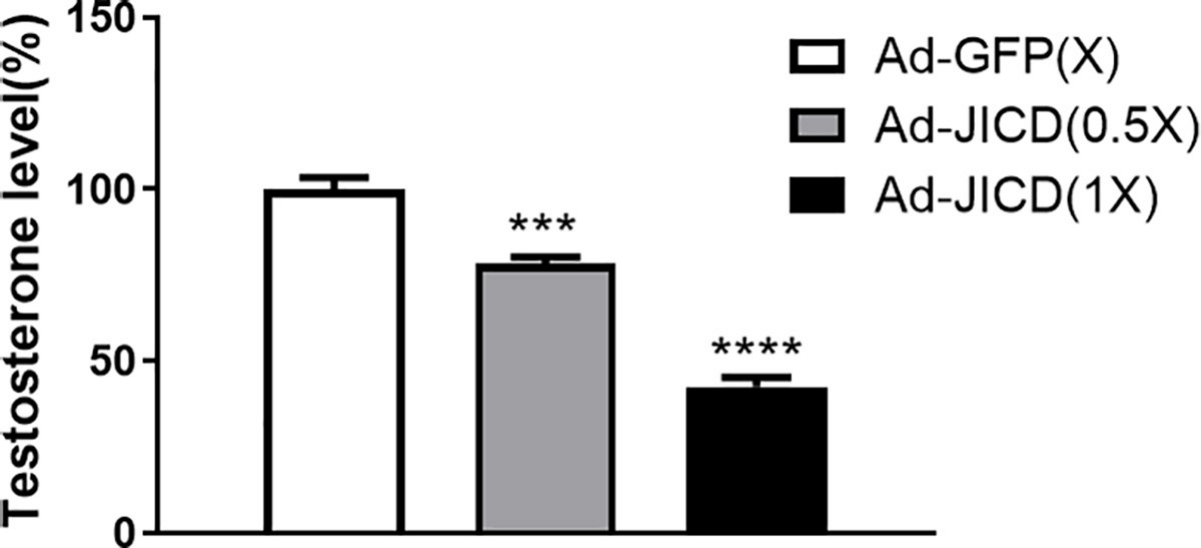
Jagged1 intracellular domain (JICD) inhibits testosterone production. Primary Leydig cells isolated from adult mice were infected with JICD-expressing adenovirus (Ad-JICD) or GFP-expressing adenovirus (Ad-GFP; control) and treated with 200 μM cAMP for 48 h. Media were collected to analyze testosterone level by radioimmunoassay. Statistical significance was evaluated by t-test relative to Ad-GFP-transduced samples. ***, P<0.001; ****, P<0.0001.

The repression of steroidogenic gene expression by JICD allowed us to test the effect of JICD on the production of testosterone in primary Leydig cells. We infected primary Leydig cells with either Ad-JICD or Ad-GFP (as a control). After stimulation of these cells with cAMP for 48 h, media were collected and the extent of testosterone production was estimated. There was more than 50% reduction in testosterone level upon Ad-JICD infection compared to the corresponding Ad-GFP control (Fig 7). Collectively, these results suggest that JICD modulates testicular steroidogenesis by controlling Nur77-induced expression of steroidogenic genes in Leydig cells.

## Discussion

Multiple signaling pathways and nuclear factors regulate testicular steroidogenesis in Leydig cells in a negative or positive manner [25, 34, 35]. The major pathway for testicular steroidogenesis is the cAMP-dependent protein kinase A signaling pathway, controlled by the pituitary gonadotropin, LH, in Leydig cells. In this study, we demonstrated the direct modulation of testicular steroidogenesis by JICD. Jagged 1, a well-known ligand of Notch1, has been predominantly reported as a regulator of progenitor Leydig cells. The roles of Jagged1 [2] and Hes1 [1] in the regulation and maintenance of the progenitor niche are also well documented. The constitutively active expression of Notch driven by *Sf1-cre* mice (*Rosa*^*Notch*^; *Sf1-cre*) does not restrict Sertoli cell differentiation, but the number of Leydig cells is acutely reduced due to differentiation failure [1]. A conditional deletion of Jagged1 is known to trigger the conversion of interstitial cells to differentiated Leydig cells [2]. These studies delineate the role of Notch signaling in the maintenance of progenitor Leydig cells. However, to the best of our knowledge, no previous study has explored the role of Notch1, Jagged1, and Hes1 in testicular steroidogenesis.

The expression of *Notch1* in the whole testis or primary Leydig cells decreases with testicular development, which coincides with its predominant function in the maintenance of progenitor Leydig cells. However, *Jagged1* expression in the whole testis and isolated testicular Leydig cells is markedly different due to the contribution from spermatids in the whole testis [29]. The constant level of *Jagged1* during Leydig cell differentiation and maturation at around 3–5 weeks could be linked to the proper balance of steroidogenic gene expression during testicular development and maturation. Similar repressive effects of CKLFSF2A and CKLFSF2B on testicular steroidogenesis have been previously reported [36, 37]. Therefore, the differential expression pattern of *Jagged1* and *Notch1* in testicular Leydig cells suggests a role of Jagged1 independent of Notch1.

LH/cAMP-mediated induction of Nur77 is considered important for the stimulation of steroidogenesis. During cAMP-mediated induction of steroidogenesis in primary Leydig cells, *Jagged1* expression is maintained in a repressed manner in contrast to the strong induction of *Nur77* expression. During the time period of 4–6 h when Jagged1 expression is restored, the first phase of Nur77-mediated induction of the expression of its target steroidogenic genes is completed. This inverse pattern of expression between *Jagged1* and *Nur77* during first 4 h suggests the contrasting action of these genes during induction of steroidogenesis by LH/cAMP signaling. The restoration of Jagged1 can be considered to fine tune the expression of Nur77 and steroidogenesis at that point of time, which is evident by consistent decrease in Nur77 expression. Since the isolated primary Leydig cells are natural cells, their sensitivity to external conditions could contribute to the steep repression of *Jagged1* expression within 2 h and markedly fast reversal to its basal level by 4–6 h. This repression and reversal pattern were similar, albeit with differential sensitivity, in primary and MA10 Leydig cells.

Jagged1 is regulated post-translationally to produce multiple active protein isoforms, including the full length Jagged1, membrane-bound C-terminal fragment, and processed intracellular domain with nuclear localization [38]. JICD displays functions independent of canonical Notch signaling. Vimentin binds to the distinct PDZ sequence of JICD and provides a force-generating mechanism to ensure efficient trans-endocytosis and Notch activation [39]. This positive correlation between Jagged1 and vimentin has been established in leucocytes and tissues of the kidney, breast, blood, heart, muscle, and testis. JICD-mediated Notch inhibition diminishes cell proliferation in neonatal cardiomyocytes and promotes maturation [11]. JICD has also been shown to regulate cell transformation and proliferation [40, 41]. The transcription factor AP1, along with the soluble form of JICD, can activate gene expression [41]. Interestingly, JICD can directly interact with NICD and is known to accelerate the degradation of NICD through the Fbw7-dependent proteasomal pathway [42]. JICD inhibition of Nur77 transactivation, implied in the present study, extends the list of Jagged1 functions independent of the conventional Notch signaling pathway.

The adenovirus-mediated overexpression of JICD in primary Leydig cells significantly repressed *Nur77* expression (Fig 6), although the level of repression was lower than that of its target genes. Nur77 expression is directly repressed through JICD-mediated inhibition of CRE-dependent promoter activity. In contrast, the target genes are subjected to a dual repressive effect; first, due to the reduced availability of Nur77, and second, on account of the repressed transactivation of available Nur77. Although we could not find the exact mechanism for the inhibition of Nur77 transactivation in the current study, we previously observed such an inhibition on steroidogenic gene promoters either by direct protein-protein interaction [37] or by reduction in the protein stability of Nur77 [43]. Testicular testosterone production is regulated by numerous biochemical components present in Leydig cells, all of which contribute partially toward the maintenance of optimal steroidogenesis. Cholesterol is transported by StAR protein into the inner mitochondria where it is converted to pregnenolone by P450scc. Pregnenolone is further catalyzed to progesterone by 3β-HSD and then to 17-hydroxyprogesterone and androstenedione by P450c17. Finally, the product is converted to testosterone by 17β-hydroxysteroid dehydrogenase (17β-HSD) [44, 45]. Therefore, the reduced expression of these steroidogenic genes by a dual repressive effect of JICD on Nur77 results in reduced testosterone production in primary Leydig cells transduced with Ad-JICD. In addition to steroidogenesis, Nur77 has been reported to be associated with Jagged1. TR3/Nur77 is a critical mediator in pathological angiogenesis [46, 47], in which TR3/Nur77-induced expression of integrins regulates the expression of Jagged1 and Dll4 [14].

In summary, we have shown for the first time that Jagged1, known for a Notch ligand, plays an independent role in testicular Leydig cells. Jagged1 undergoes proteolytic processing similar to Notch1 to release JICD, which can translocate to the nucleus and regulate Nur77 transcription. It also inhibits Nur77 transactivation, further repressing the expression of steroidogenic genes. This suppressive regulation of steroidogenic gene expression by JICD results in reduced testosterone production. Altogether, Jagged1, modulates steroidogenesis in testicular Leydig cells, fine-tuning the level of testicular testosterone during testis development.

## Material and methods

### Animals and treatment

ICR and C57BL/6 mice were purchased from a commercial supplier (Dae-han Laboratories, Daejeon, Republic of Korea) and maintained at 22±3°C with a humidity level between 40% and 60% in a 12:12 h light-dark cycle with food and water *ad libitum*. The selection of mouse of a certain age was based on previous studies that reported the development of adult Leydig cells [48]. Total 74 mice were used; 12 mice of 3 weeks, 9 each of 4 and 5 weeks and 54 mice of 6 weeks. The mice were sacrificed by CO_2_ asphyxiation followed by cervical dislocation, and then their testes were dissected. Ethical treatment of the animals was performed according to National Institutes of Health standards. All animal procedures were approved by Institutional Animal Care and Use Committee (AICUC) of Chonnam National University (Permit Number: 2012–44)

### Plasmids

The mammalian expression vectors, pCDNA-Flag-JICD and pCDNA-Nur77, and reporter plasmids, NBRE-luc, Nur77 promoter-luc, StAR-luc, Cre-luc, 3β-HSD-luc, and P450c17-luc, were described previously [49–53].

### Cell culture and purification of primary Leydig cells

The MA-10 mouse Leydig cell line was a generous gift from Dr. Mario Ascoli (University of Iowa, Iowa) [54]. MA-10 cells were maintained in RPMI 1640 medium (containing 25 mM HEPES) supplemented with 15% horse serum and antibiotics [55]. All cells were cultured at 37°C in a 5% CO_2_ atmosphere. Primary mouse Leydig cells were isolated as previously described [43]. Briefly, testicular cells were dispersed by treating decapsulated mouse testes with collagenase type I (0.25 mg/mL, Life technologies Corporation, New York, USA), which was maintained at room temperature for 20 minutes with gentle shaking and were tapped at every 5 minutes to disperse the testicular tubules. Interstitial cells were prepared by filtering the testicular cells with a 40 μm cell strainer (BD Biosciences, San Jose, CA). The cells were then washed twice with M199 medium, collected after centrifugation and plated in RPMI 1640 medium (containing 25 mM HEPES) supplemented with 15% horse serum and antibiotics. When needed, Leydig cells were activated by treating 200 μM cAMP for the indicated time period.

### Adenovirus infection

Primary Leydig cells from 6-week-old mice were plated in RPMI 1640 medium (25 mM HEPES) containing 5% charcoal stripped serum and antibiotics. After 24 h, cells were infected with Ad-GFP or Ad-JICD and kept for 16 h further. Culture media was then replaced and cells were incubated for 48 h including 200 μM cAMP treatment for last 4 h. After collection, cells were washed with PBS and processed for RNA isolation. Further qPCR was performed with total RNA for quantification of gene expression.

### Radioimmunoassay (RIA)

To measure testosterone levels, serum-free culture medium of primary mouse Leydig cells, infected with Ad-GFP or Ad-JICD and treated with cAMP for 48 h, was collected. This medium was separated by centrifugation at 10000g for 5 min at 4°C and then stored at -70°C for performing testosterone assays. Testosterone concentration was measured by RIA as described previously [56]. The inter- and intra-assay coefficients of variation for the testosterone estimation were 8.7% and 9.3%, respectively [57].

### Transfection assay

MA-10 cells were transiently transfected using Lipofectamine 2000 (Life Technologies Corporation, Carlsbad, CA, USA) transfection reagent, according to the manufacturer’s instructions. For luciferase assay, MA-10 cells were plated in media containing 5% charcoal-stripped serum 24 h prior to transfection and transfected with expression vectors, a reporter gene, and the control *lacZ* expression plasmid, pCMV (Clontech, USA) or pSV-β-gal (Promega Corporation, Wisconsin, USA). The total amount of DNA was maintained by adding the pcDNA3 empty vector. Luciferase and β-galactosidase activities were assayed as described previously [58]. The measured luciferase activity was normalized to *lacZ* expression. For siRNA experiments, CREB siRNA 5'-UACAGCUGGCUAACAAUGG-3' and a negative control siRNA (catalog no. 1003, Bioneer Corporation, Daejeon, Republic of Korea) were used.

### Quantitative PCR

Total RNA was prepared from mouse testes, primary Leydig cells, and MA-10 cells using TRI reagent (Molecular Research Center, Inc. Cincinnati, USA) according to the manufacturer’s instructions. Reverse transcription reactions were performed with 2 μg of total RNA using M-MLV reverse transcriptase (Promega). Quantitative PCR was performed with TOPreal qPCR 2X PreMIX SYBR green with high ROX (Enzynomics, Daejeon, Republic of Korea) using StepOnePlus^TM^ Real-Time PCR System (Applied Biosystems, Carlsbad, CA) according to the manufacturer’s instructions. The primers used for real-time PCR are listed in S1 Table.

### Statistical analysis

To identify significant differences, data were analyzed using GraphPad Prism version 8.0 (San Diego, CA) Single comparisons between two experimental groups were analyzed using an unpaired Student’s *t*-test or one-way ANOVA. Data are presented as the mean ± SEM of at least three independent experiments. For all statistical analyses, P < 0.05 was considered significant.

## Supporting information

S1 Table List of gene-specific oligomers used for quantitative PCR. (DOCX)
