# Supplementary material for: Jagged1 intracellular domain modulates steroidogenesis in testicular Leydig cells
Source: PLoS One. 2020 Dec 30;15(12):e0244553. doi: 10.1371/journal.pone.0244553 (PMC7773251; doi:10.1371/journal.pone.0244553)
Supplement: S1 Table — (DOCX) [file pone.0244553.s001.docx]

**Gene specific oligomers used for Quantitative PCR**:

| Nur77 Sense | 5’-CTC GCC ATC TAC ACC CAA CT-3’ |
| --- | --- |
| Nur77 Antisense | 5’-AGC CTT AGG CAA CTG CTC TG-3’ |
| StAR Sense | 5’-TGT CAA GGA GAT CAA GGT CCT TG-3’ |
| StAR Antisense | 5’-CGA TAG GAC CTG GTT GAT GAT-3’ |
| P450c17 Sense | 5’-TGG CCC CCT TGC TCA TCC CA-3’ |
| P450c17 Antisense | 5’-TCG GGG ACC AGC TCC GAA GG-3’ |
| 3β-HSD Sense | 5’-ACT GCA GGA GGT CAG AGC T-3’ |
| 3β-HSD Antisense | 5’-ATG GTC TGC CTG GGA ATG AC-3’ |
| P450scc Sense | 5’-CTG CCT CCA GAC TTC TTT CG-3’ |
| P450scc Antisense | 5’-TTC TTG AAG GGC AGC TTG TT-3’ |
| GFP Sense | 5’-GTC GTC CTG CTT CAT GTG G-3’ |
| GFP Antisense | 5’-GGC GAG GAG CTG TTC ACC-3’ |
| Jagged1 Sense | 5’-TGA CAT GGA TAA ACA CCA GCA-3’ |
| Jagged1 Antisense | 5’-GCA GCC CAC TGT CTG CTA TAC-3’ |
| GAPDH Sense | 5’-ATC ACC ATC TTC CAG GAG CGA G-3’ |
| GAPDH Antisense | 5’-GAG ATG ATG ACC CTT TTG GCT CC-3’ |
| β-actin Sense | 5’-TTC TAC AAT GAG CTG CGT GTG-3’ |
| β-actin Antisense | 5’-GGG GTG TTG AAG GTC TCA AA-3’ |
